# The importance of graph databases and graph learning for clinical applications

**DOI:** 10.1093/database/baad045

**Published:** 2023-07-10

**Authors:** Daniel Walke, Daniel Micheel, Kay Schallert, Thilo Muth, David Broneske, Gunter Saake, Robert Heyer

**Affiliations:** Bioprocess Engineering, Otto von Guericke University, Universitätsplatz 2, Magdeburg 39106, Germany; Database and Software Engineering Group, Otto von Guericke University, Universitätsplatz 2, Magdeburg 39106, Germany; Database and Software Engineering Group, Otto von Guericke University, Universitätsplatz 2, Magdeburg 39106, Germany; Multidimensional Omics Analyses Group, Leibniz-Institut für Analytische Wissenschaften—ISAS—e.V., Bunsen-Kirchhoff-Straße 11, Dortmund 44139, Germany; Section eScience (S.3), Federal Institute for Materials Research and Testing (BAM), Unter den Eichen 87, Berlin 12205, Germany; Infrastructure and Methods, German Center for Higher Education Research and Science Studies (DZHW), Lange Laube 12, Hannover 30159, Germany; Database and Software Engineering Group, Otto von Guericke University, Universitätsplatz 2, Magdeburg 39106, Germany; Multidimensional Omics Analyses Group, Leibniz-Institut für Analytische Wissenschaften—ISAS—e.V., Bunsen-Kirchhoff-Straße 11, Dortmund 44139, Germany; Faculty of Technology, Bielefeld University, Universitätsstraße 25, Bielefeld 33615, Germany

## Abstract

The increasing amount and complexity of clinical data require an appropriate way of storing and analyzing those data. Traditional approaches use a tabular structure (relational databases) for storing data and thereby complicate storing and retrieving interlinked data from the clinical domain. Graph databases provide a great solution for this by storing data in a graph as nodes (vertices) that are connected by edges (links). The underlying graph structure can be used for the subsequent data analysis (graph learning). Graph learning consists of two parts: graph representation learning and graph analytics. Graph representation learning aims to reduce high-dimensional input graphs to low-dimensional representations. Then, graph analytics uses the obtained representations for analytical tasks like visualization, classification, link prediction and clustering which can be used to solve domain-specific problems. In this survey, we review current state-of-the-art graph database management systems, graph learning algorithms and a variety of graph applications in the clinical domain. Furthermore, we provide a comprehensive use case for a clearer understanding of complex graph learning algorithms.

**Graphical abstract**

**Figure:**
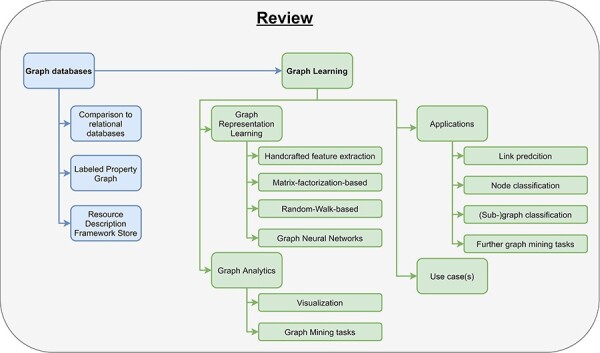

**Key points**

- Review current graph databases and graph learning algorithms.
- Review graph learning applications in medicine.
- Clearer understanding by introducing a medical use case for applying graph learning algorithms.

## Introduction

**Figure 1.**
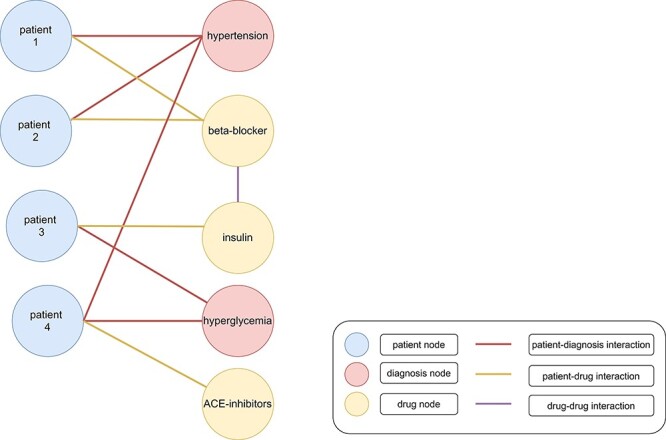
Heterogenous graph with clinical data. Patients (blue nodes) are connected with diagnoses (red nodes) with a red edge and drugs (yellow nodes) with a yellow edge. Interactions between different drugs are represented as a purple edge. Patients 1 and 2 were diagnosed with hypertension. Therefore, beta-blockers were administered as treatment. Patient 3 was diagnosed with hyperglycemia and cured with insulin. However, patient 4 was diagnosed with hypertension and hyperglycemia (199). The administration of beta-blockers and insulin would lead to negative side effects because of an increased risk for hypoglycemia. Therefore, angiotensin-converting enzyme (ACE) inhibitors might be administered as treatment (200).

**Figure 2.**
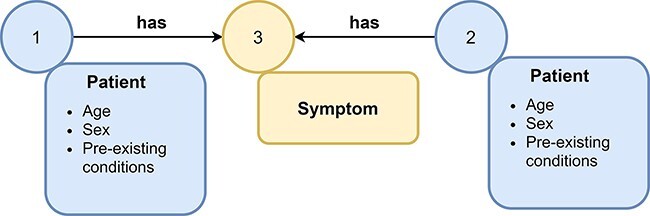
Example of a graph. A graph consists of nodes (represented as circles) and edges (represented as arrows) that connect nodes with each other. Nodes and edges can have labels, e.g., symptom or patient, and additional attributes. Attributes (often referred to as features) contain additional information about nodes or edges, e.g., the age and sex of a patient node.

The amount and the complexity of clinical data is continuously increasing (1). Ongoing digitalization in the biomedical domain makes big clinical data sets more available for storage and subsequent analysis. The insights hidden in this data offer the potential for improved treatments of patients, e.g., by more accurate and faster diagnoses, examination of adverse effects and discovering of new drugs for specific targets (2). However, the rapidly increasing data present serious problems in storing and processing these data appropriately (3). Traditionally, relational databases store such data in a tabular structure and use SQL (Structured Query Language) for querying them. Recently, non-relational stores, so-called NoSQL databases, became popular to handle the shortcomings of relational databases for storing and querying big data (e.g., less flexible data schemas). NoSQL databases include key-value stores, wide-column stores, document stores and graph stores (2). Graph databases use graph stores and provide efficient entity traversals, i.e., they are ideal for handling interconnected (i.e., entities with one or multiple relationships/interactions between each other) and heterogeneous (i.e., different kind of entities) data, such as clinical data (e.g., patients with similar laboratory results having similar treatments in a graph with connections based on the laboratory results and treatments of each patient (Figure 1)) (3). Graph stores represent data in form of graphs consisting of nodes (also called vertices) and edges (also called links or relations), which connect nodes with each other (4). Nodes, edges, or complete graphs can have labels and features (also called attributes) (5). For example, a graph containing patients and symptoms might use node labels to distinguish between these two types of nodes (Figure 2). Additionally, a patient might have further information, e.g., sex, age and pre-existing conditions, stored as node features.

A large graph that represents one fact as a triplet consisting of a head-entity (node), a relation (edge) and a tail-entity (node) is called a knowledge graph (6). While we would like to stick to this definition of Knowledge Graphs for simplicity, there exist some further definitions of knowledge graphs (7). Knowledge graphs have several different applications, including Health Knowledge Graphs (8–10), Biological Knowledge Graph (11, 12), Knowledge Graphs for Covid-Research (13–15) and many others (16–18). Graph databases are already used in several other applications, e.g., social networks (19–22), recommendation systems (23, 24) and fraud detection (25, 26). Several different graph database management systems (DBMSs) have been proposed, e.g., Neo4J (27), NebulaGraph (28), TigerGraph (29), DGraph (30), ArangoDB (31) and many more (32). They are distinguished from each other in their query language, their data storage model [i.e., labeled property graph (LPG) or resource description framework (RDF)], the availability of access controls, the supported programming languages and their license. One of the currently most popular graph databases, Neo4j, uses native graph storage and processing. The query language Cypher is used to query data and Neo4j offers different graph analytics algorithms in their Graph Data Science Library (27).

After choosing a graph DBMS for the desired use case, users need to design a graph data model to store their data in the graph database. While constructing an appropriate data model, users typically face the following challenges:

- Which data should be stored as separate nodes, edges, or graphs?
- Which data should be stored as node/link/graph attributes?
- What labels should the stored nodes/edges/graphs have?
- How to define attributes to make them accessible for further processing steps?

Unfortunately, there is no ‘one size fits all’ solution for these questions. It depends on the data, i.e., whether the data is numeric (discrete/continuous) or categorical (nominal/ordinal), and the intended use case, i.e., what questions should be answered with the data. If the goal is to predict interactions between different drugs, we would model drugs as nodes and known interactions as edges (drug–drug interaction graph) (33–35). However, if the goal is to predict properties of different drugs, we might represent each drug agent in its chemical structure as an individual graph with nodes as atoms and atomic bonding as edges (36–40).

After storing the data in an appropriate data model, the next goal is to analyze the graph data. This is where graph learning becomes important. Graph learning is the application of machine learning techniques on graph data, i.e., it simplifies complex interconnected data to draw conclusions and answer specific questions (41). It is distinguished from other machine learning techniques by making use of the graph structure (nodes and the relations between them). Graph learning consists of two parts, graph representation learning and graph analytics. Graph representation learning is used to reduce the dimensionality of the input graph, which might include millions or even billions of nodes and edges (42). There are several graph representation learning techniques, which can be divided into (i) matrix factorization-based approaches, (ii) random walk-based approaches and (iii) graph neural networks (GNNs). The output of graph representation learning is a set of low-dimensional embedding vectors (i.e., one for each node), or a single embedding (i.e., for the entire graph) that can then be used in graph analytics for graph mining tasks, or visualization tasks. The most commonly used graph mining tasks for analytics are node classification, link prediction and graph classification (42). These tasks are already used in several clinical real-world use cases, including predicting interactions between different drugs (43) and diagnosing patients based on their medical history (44, 45). Graph learning is not only a promising technology for decision-support systems and drug discovery, but also for generating new knowledge through Knowledge Graph completion (46, 47). Furthermore, modern hardware like GPUs and FPGAs can further accelerate analyzing large amounts of graph data by parallelizing some calculations (48, 49).

### Organization

In this paper, we will define and explain graph databases and review several different graph databases (Section 2). Then, we illustrate the concept of graph learning, review the most important graph learning algorithms, and explain their limitations (Section 3). Finally, we show some already existing applications for graph learning in the clinical domain (Section 4.1–Section 4.4).

### Our contributions

**Figure 3.**
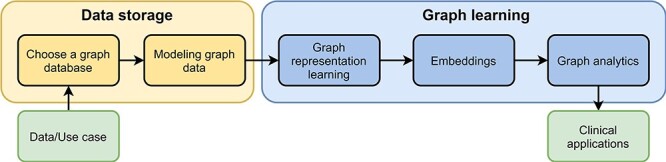
Pipeline for processing graph data. The pipeline consists of two parts, data storage and graph learning. As input users have data and a specific use case. After they have chosen a graph database, they need to design a graph data model to store their data appropriately (data storage). In the second part, high-dimensional graph data are reduced to obtain low-dimensional embeddings using graph representation learning. These embeddings are used in graph analytics for several different clinical applications, e.g., predicting interactions between drugs.

There already exist some review articles about graph databases and graph learning comparing many different approaches and embedding methods (32, 42, 50–53). Besides them, there are few publications about either graph databases in the biomedical domain or graph representation learning in bioinformatics (51, 54, 55). To the best of our knowledge, this is the first survey incorporating a complete pipeline from data storage, over graph learning to real-world applications in clinical domains (Figure 3). Especially for beginners in this field, our work helps in getting started with graph databases and provides a comprehensive introduction for processing big clinical data. While this review primarily focuses on clinical applications, the concepts introduced in this work can also be applied to many other applications, e.g., recommendation systems and fraud detection.

## Graph databases

In large-scale machine learning applications, data storage is as important as the machine learning algorithms that learn patterns in the data (56, 57). Data must be securely stored, especially for clinical applications, readily available for training of machine learning algorithms and remain updateable with new information. Databases are organized as collections of data that, depending on their DBMS support different types of data and allow for access patterns that are beneficial in different applications. Historically, relational database systems have been used to store the majority of data in production settings. In this section, the journey from classic relational databases to graph databases will be explored. First, the traditional relational database management systems will be introduced, as well as the challenges that arise from joins in relational databases (Section 2.1). Secondly, it will be shown how graph databases represent data and how the graph data representation can partially overcome the challenges of relational databases (58) (Section 2.2 and Section 2.3). Finally, we will explain why we need graph query languages and name the most important representatives (Section 2.4).

### Relational Database Management System (RDBMS)

In relational database management systems, single records are represented as rows in a defined table structure. The table structure is based on columns that represent the attributes of the data. Each record can be identified by a primary key. A relation can be represented by using the identifiers of records as foreign keys. These keys can be used in join tables to identify a row in one table with a row in another table.

Primary keys are a column or set of columns that uniquely identify a row in a table. Foreign keys are a column or set of columns that identify a row in another table.

SQL is the standard language for accessing and manipulating records in an RDBMS Most relational databases support SQL and it is the standard for data manipulation in RDBMS (58, 59).

#### Joins

Joins are query operations on a relational database that allow for the retrieval of multiple records from different tables according to a join condition. Joins therefore link these records from the RDBMS together based on the join condition. Join conditions are logical comparisons of fields or keys between tables. Joins can therefore be used for combined retrieval of related entries from two or more tables in the RDBMS. This makes Joins the preferred method for querying data based on a relationship between the elements in the table that is encapsulated in the logical condition. Joins can be separated based on the way the data is retrieved from the joined tables.

**Figure 4.**
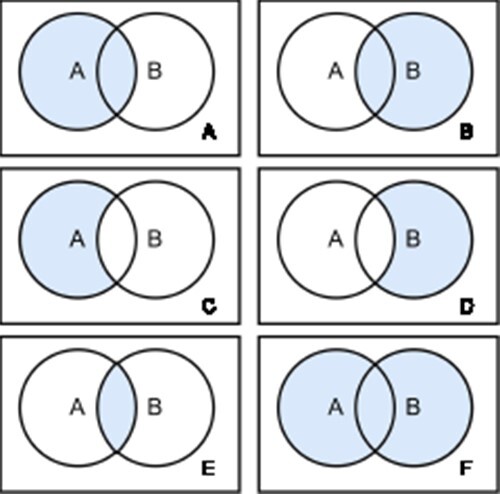
Join Types. There are six different join types: left Outer Join (A), right Outer Join (B), left Outer Join with Null (C), right Outer Join with Null (D), inner Join (E) and full Outer Join (F). Each circle represents a table and the blue color highlights the retrieved data from the joined tables.

There are two types of joins defined in SQL: inner joins and outer joins. Inner joins return rows from both tables that match the join condition. Outer joins return rows from the one table that does not match the join condition (Figure 4). Therefore, left outer joins return all rows from the left table and all rows from the right table that satisfy the join condition. Conversely, right outer joins return all rows from the right table and all elements from the left table that satisfy the join condition. Full outer joins return all rows of both sides pivoted on the field in the join condition (60).

References by foreign key can be resolved by the SQL JOIN operation. Many-to-many relations in relational DBMSs require multiple JOIN operations. Relational DBMSs struggle to integrate new ad-hoc relations between records in the table structure. New relations that are introduced into the existing relational database require join tables that limit the performance of the database. However, relational databases provide materialized database views that store results from SQL operations like JOINS in a new materialized table (61). After the creation of this materialized table, one can directly query these data without requiring any JOIN operations. Thereby, views facilitate querying connected data from multiple different tables and increase the query performance. Furthermore, specific columns in this new table can be indexed to further accelerate potentially computational expensive search, comparison and filtering operations (62). Another direction to overcome performance issues in RDBMS is to use specific data structures tailored to the desired use case. One example might be the integration of more information in a single table (e.g., denormalization to WideTables (63)) to prevent potentially expensive JOIN operations.

### Graph Database Management System (GDBMS)

NoSQL databases are non-relational databases. Contrary to relational databases, these databases do not represent their data in a table structure. The field of NoSQL databases includes a variety of databases with different data models; these include but are not limited to document-oriented databases, key-value databases, wide-column databases, RDF stores and native graph databases (2, 58).

Graph database management systems (GDBMS) are NoSQL databases that use graph structures for semantic queries with nodes, edges and properties to represent and store data and the relationships exhibited by the data. A key concept of the system is the graph, which can be modeled in various ways. The direct relationships of data in the graph allow data in the store to be linked together directly and, in many cases, retrieved with one operation. These retrievals can be powerful when the data is interconnected. A graph database can be used to represent any kind of data that has relationships between data elements. Therefore, graph databases are often used in applications where the underlying data has a lot of relationships between items, or where the relationships between data items are more important than the individual items.

#### Native graph databases

A database that models graph data can be implemented in any NoSQL data model and even relational databases (32). Different data representations therefore require different concepts to represent nodes, edges, labels and properties contained in a graph. Graph databases exist that are built on tuple stores, wide-column databases, key-value databases, document databases and relational databases.

However, the LPG and RDF are two data models that implement a graph natively for utilization as a database.

#### Differences between relational and graph databases

**Figure 5.**
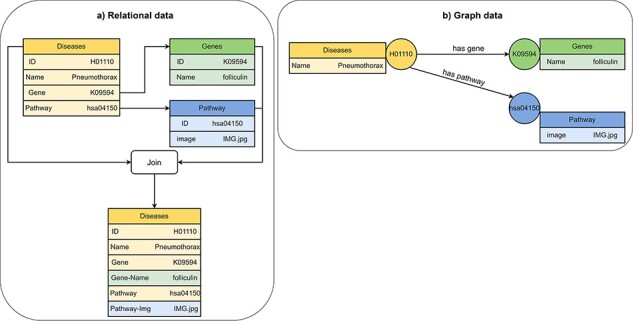
Visualization of (A) relational data and (B) graph data of disease, gene and pathway entities in KEGG (201). A disease references to a pathway entity and to a gene entity. For receiving information from multiple tables in a relational database, we must perform join operations on the tables. In a graph database, we traverse edges to receive information from multiple nodes.

Compared to graph databases, relational databases perform better in respect to query efficiency and data modeling for storing data that can be easily normalized into a tabular format (58). Graph databases are better suited for storing and accessing data where most data is interconnected, such as social data, location data, network data and biomedical data (Figure 5) (64). Relational databases are better for online transaction processing (OLTP) applications because relational databases are better at supporting transactions and maintaining data integrity. Relational databases are typically more expensive to maintain and to scale than graph databases because inserting new data elements with relationships to old data elements requires join operations for the integration into the existing tabular data model. Many different data models can be used to implement graph databases. The most important native Graph data models are RDF stores (65) and LPG (32).

### Graph data models

#### Graph model

A graph G(V, E) is composed of an ordered pair of two disjoint sets: vertices V (also referred to as nodes) and edges (or links) E (32). In information theory, a graph is a representation of a set of objects and relationships between them. Graphs can be used to model a wide variety of structures, including networks, hierarchies, ontologies and other forms of interconnected data. In native graph databases, a graph can be represented as either an LPG or as RDF triples (65).

#### Labeled Property Graph (LPG)

**Figure 6.**
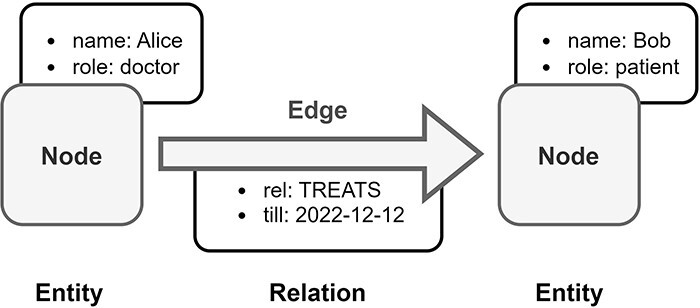
Overview of Labeled Property Graph (LPG). In this example, medical data is transformed into an LPG format. Alice is a doctor and Bob is a patient, both are represented as nodes in the LPG. The doctor–patient relationship between Alice and Bob is modeled by a directed edge in the LPG with an assigned property, which is the end date of Bob’s treatment by Alice.

Property graphs use a node-edge-property model, where each node represents an entity, each edge represents a relationship between two entities, and each property represents an attribute of an entity or relationship (Figure 6). The property graph model is based on the classical graph model, but introduces labels to vertices, edges, and properties. Labeled vertices and edges can represent different classes of data. Properties augment the vertices and edges with key-value pairs, where the key identifies the property and the value represents the feature of the property (32, 66).

#### Neo4j

Neo4j is a GDBMS that uses native graph storage and processing. Neo4j uses the LPG data model and the Cypher query language. Neo4j graphs are directed however this limitation can be overcome at query time (27, 32, 67).

#### SparkSee

Sparksee is an LPG graph database management system. Sparksee allows directed and undirected graphs. Sparksee uses the OpenCypher query language (32, 68).

#### TigerGraph

TigerGraph is a property graph database, which supports the LPG model. TigerGraph uses a SQL-like query language (GSQL). TigerGraph offers built-in MapReduce operations and parallelism (29, 32).

#### Resource Description Framework (RDF) stores

**Figure 7.**
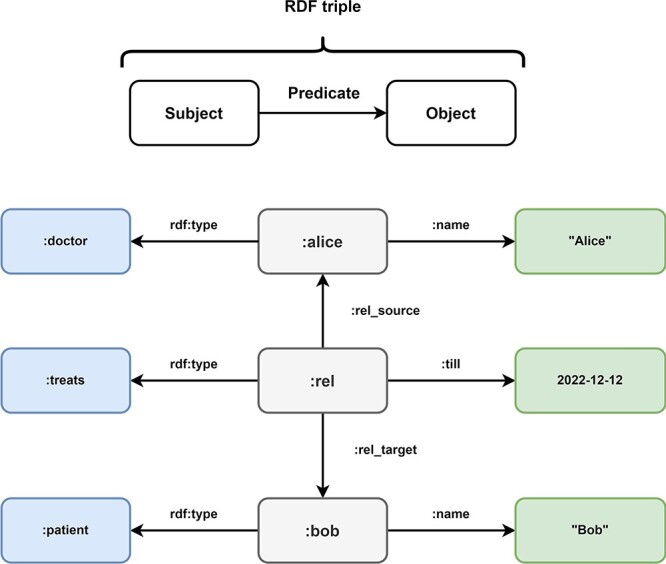
Overview of Resource Description Framework (RDF). Every information in the Resource Description Framework is organized as a triple and every triple follows a subject predicate object structure. In this example, the medical data of Figure 5 is transformed into the RDF format. The subject:alice has the predicate rdf:type with the object:doctor and the predicate:name with object ‘Alice’. These RDF triples represent the node of doctor Alice in the LPG. The directed nature of the edge TREATS in the LPG is represented by the RDF triples (:rel,:rel_source,:alice) and (:rel,:rel_target,:bob). The properties of the edge are represented by the triples (:rel, rdf:type,:treats) and (:rel,:till, 2022–12-12). Bob is represented by the triples (:bob, rdf:type,:patient) and (:bob,:name, “Bob).

The RDF (69) was first published by the World Wide Web Consortium (W3C) in 1997 as a collection of specifications for the representation of information. The RDF was revised in 2014 to version 1.1. The goal of the RDF is the standardization and simplification of the exchange of ontologies. Ontologies are sets of terms and the relationships between them. To facilitate this goal, RDF triples use a subject-predicate-object graph model, where each triple represents a relationship between two items in the graph (Figure 7). Subjects are Uniform Resource Identifiers (URIs) or blank nodes, objects can be URIs, literals or blank nodes and predicates are URIs. The collection of these information triples forms a collection that is often referred to as a triple store. RDF triples are a standard format for linked data (32, 70).

### Graph query languages

Besides their data model, databases can be classified by their query language. A query language is used for the retrieval and access to data in databases. These query languages facilitate the access, manipulation, and creation of data in the database. In graph databases, the data model also influences the used query language. While most RDF databases rely on the SPARQL Protocol and RDF Query Language (SPARQL) for data access and manipulation, most LPG databases employ Cypher, Gremlin, or GSQL as their query language (71). Tables 1–3 provide an overview how to create a graph (Table 1), create/delete nodes (Table 2), and create/delete edges (Table 3) in different query languages.

**Table 1.** Overview of how to create graphs using different graph query languages

Query language	Create graph
Cypher	CREATE DATABASE graphName
GSQL	CREATE VERTEX nodeType (PRIMARY_ID idName idType, feature1 featureType) CREATE UNDIRECTED EDGE edgeType (FROM nodeType, TO nodeType, featureValue featureType) CREATE GRAPH graphName (nodeType, edgeType) USE GRAPH graphName
Gremlin	graphName = TinkerGraph.open().traversal()
SPARQL Update	CREATE GRAPH IRIref

**Table 2.** Overview of how to create and delete nodes using different graph query languages

Query language	Insert nodes	Delete nodes
Cypher	CREATE (n:nodeType {feature1: value})	MATCH (n:nodeType {feature1: value}) DELETE n
GSQL	INSERT_INTO nodeType VALUES (idValue, featureValue)	CREATE QUERY queryName() FOR GRAPH graphName { N = {nodeType.*}; DELETE n FROM N:n WHERE n.feature1 == featureValue; } RUN QUERY queryName()
Gremlin	graphName.addV().property(id, idValue).property(feature1, value).next()	graphName.V().hasId(idValue).drop()
SPARQL Update	INSERT DATA {GRAPH IRIref {URI_subject URI_predicate value}}	DELETE DATA {GRAPH IRIref {URI_subject URI_predicate value}}

**Table 3.** Overview of how to create and delete edges using different graph query languages

Query language	Insert edges	Delete edges
Cypher	MATCH (m:nodeType), (n:nodeType) WHERE m.feature1 = featureValue AND n.feature1= featureValue CREATE (m)-[e:edgeType]->(n)	MATCH (m:nodeType)-[e:edgeType]-> (n:nodeType) DELETE e
GSQL	INSERT INTO edgeType (FROM, TO, feature1) VALUES (startNode nodeType, endNode nodeType, featureValue)	CREATE QUERY queryName() FOR GRAPH graphName { N = {nodeType.*}; DELETE e FROM N:n -(edgeType:e)- nodeType:m WHERE n.feature1 == featureValue; } RUN QUERY queryName()
Gremlin	graphName.addE(‘edgeLabel’).from(startNode).to(endNode).property(id, idValue).property(feature1, value)	graphName.E().hasId(idValue).drop()

#### Cypher

Cypher is Neo4j’s own declarative query language (72). Its main building blocks are ‘patterns’. These patterns are constructed as nodes in squared brackets and connecting edges as arrows (66).

#### GSQL

GSQL is TigerGraph’s high-level graph querying and update language with a declarative semantic. It is closely modeled after SQL from relational databases (SELECT-FROM-WHERE as the core building block). Therefore, GSQL is compatible with SQL (73, 74).

#### Gremlin

Gremlin is a graph traversal language introduced in 2009. Gremlin is maintained and developed by Apache TinkerPop (75). Gremlin supports imperative and declarative querying of graph data in graph databases. Apache TinkerPop is a graph computing framework for both OLTP and online analytical processing applications (66, 75).

#### SPARQL

SPARQL is a semantic query language introduced in 2008. SPARQL is able to retrieve, manipulate and store data in the RDF format (66, 76).

## Graph learning

After loading all data in the chosen graph database in an appropriate data model, data analysis becomes important. Graphs are non-Euclidean data structures (77), i.e., nodes in a graph can have a complex topological structure and do not have a natural order like pixels in a picture or words in a sentence. In this work, a topologically complex structured graph is defined as a graph with a large number of nodes (thousands, millions or even billions of nodes) which are connected by many edges based on one or multiple relationships. Several traditional approaches (Section 3.1) were proposed within the last decades to analyze graphs with different measures, statistics, or kernels (41, 78–85). They represent the pioneer works for analyzing graph data, but they are transductive (i.e., they can only learn from observed nodes) and computationally inefficient. Recently, machine learning on graphs (graph learning) has become popular. It consists of two parts, graph representation learning (Section 3.2) and graph analytics (Section 3.3). Graph representation learning transfers a given topologically complex structured input graph into a more accessible format. In graph analytics, graph learning uses this format for several tasks including visualization, classification, and prediction tasks. Table 4 lists advantages and disadvantages of traditional approaches, matrix-factorization-based methods, random-walk-based methods, and GNNs.

**Table 4.** Advantages and disadvantages of different categories of graph learning approaches used for analyzing graphs

Category	Advantages	Disadvantages
Traditional approaches (manual feature extraction)	Easy to use and understand	Require careful, hand-engineered statistics/measures
		Time-consuming and expensive design (35)
Matrix-factorization-based	Capture global structure (44)	Cannot use features, no parameter sharing, transductive, deterministic measure of neighborhood overlap (35, 36), high time and memory cost (44)
Random-walk-based	Stochastic measures of neighborhood overlap (35)	Cannot use features, no parameter sharing, transductive (35)
Graph neural networks	Most can use features,	Low interpretability, risk of over-smoothing, under-reaching and over-squashing (35, 36, 75, 77)
	Most are performant,	
	Can process large and complex data,	
	Some are inductive (35, 74)	

### Traditional approaches (manual feature extraction)

Traditional approaches can be divided into approaches for node-level, graph-level, and edge-level feature extraction. Machine learning classifiers (e.g., logistic regression) use the resulting features for making predictions. Node-level features can encode information like the number of neighbors of each node (node degree), a node’s importance in a graph (node centrality), and a node’s neighborhood cluster density (clustering coefficient) (86). Graph-level features can extract information by aggregating statistics/features from all nodes within a graph, iterative neighborhood aggregation (Weisfeiler-Lehman kernel), or counting the occurrence of different small subgraph structures (graphlet kernel) (78–82, 86). Finally, edge-level feature approaches extract features by counting the number of neighbors that two nodes share (local neighborhood overlap detection) or counting the numbers of paths of all length between two nodes (global neighborhood overlap detection) (41).

### Graph representation learning

**Figure 8.**
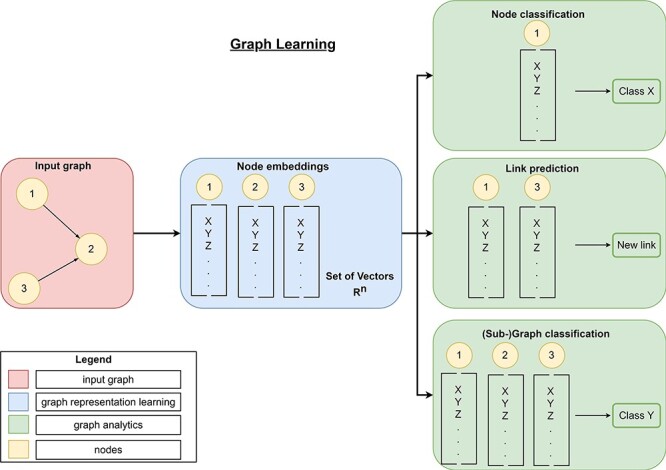
Overview of graph learning (machine learning on graphs). Graph learning can process a high-dimensional input graph (red rectangle) using graph representation learning (blue rectangles) and graph analytics (green rectangles). Graph representation learning allows the generation of low-dimensional node embeddings using graph representation approaches (manual feature extraction, matrix-factorization-based, random-walk-based, or graph neural networks). These embeddings represent feature information and topological information about a node efficiently. Then, graph analytics use these low-dimensional embeddings for graph mining tasks like link prediction to predict new links, node classification to classify nodes or (sub-)graph classification to classify entire (sub-)graphs.

Traditional approaches require careful, hand-engineered statistics and measures, and their design is time-consuming and expensive (42). Graph representation learning provides a more flexible approach and aims to find efficient task-independent representations of nodes in a graph or subgraph. Therefore, these algorithms aggregate information of each individual node as well as information from its local neighborhood into a single vector (embedding). Depending on the task (node-level, edge-level, or graph-level task), these embeddings are either directly (node-level tasks) passed to state-of-the art machine learning (SOTA-ML) algorithms or the embeddings are further aggregated (edge-level task or graph-level task) and then passed to SOTA-ML algorithms for final predictions (graph analytics, Figure 8). Besides these so-called graph mining tasks, the resulting embeddings can also be used for visualizing data (Section 3.3). Modern graph representation learning can be divided into three broad categories—matrix factorization-based methods, random walk-based methods and GNNs.

#### Matrix-factorization-based methods

Matrix-factorization-based methods represent node connections in the form of a similarity matrix (e.g., adjacency matrix, Laplacian matrix, k-step transition probability matrix) and use matrix factorization for generating embeddings (50, 53). Intuitively, they aim to learn embeddings so that the multiplication of node embedding i and j approximates their similarity (42). The factorization-based approaches vary based on the matrix properties (53). The most common representatives of matrix-factorization-based approaches include locally linear embeddings (LLE) (87), Laplacian Eigenmaps (88), Graph Factorization (89), HOPE (90) and GraRep (91, 92).

#### Random-Walk-based methods

**Figure 9.**
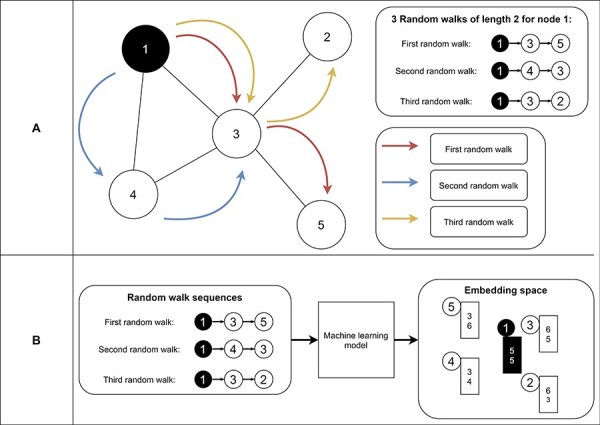
Example of random walks on a graph (A) and the transformation into node embeddings (B). In (A), three random walks with length two are sampled from node 1 to receive node sequences as a result. These node sequences represent the neighborhood of node 1. Then, we aim to find node embeddings such that nodes that co-occur often on random walks are embedded closer to each other (B). Note that the sequences generated from the random walks and the numbers for representing the final node embeddings are fictitious to make the embedding generation clearer.

Random-walk-based approaches learn embeddings based on random walk statistics (42). Random walks are very powerful shown in several applications like Google’s page rank algorithm (93). A random walk collects a series of nodes based on their connection (Figure 9A). It starts from a specific node and picks the next node randomly from the node’s neighborhood. The latter step is repeated n times, where n represents the length of the random walk. As a result, we receive for each node a set of node sequences which represent the neighborhood of the node. Finally, we use these neighborhoods in an optimization function to find node embeddings (i.e., vectors) so that nodes on the same sequence are embedded close to each other (i.e., have similar vectors) (Figure 9B). Thereby, random-walk-based approaches allow measuring the similarity of a node based on its sampled neighborhood (42). Random-walk-based approaches include techniques like DeepWalk (94) and node2vec (95). DeepWalk uses successfully established deep learning techniques of natural language processing for graph analysis. Short random walks generate latent representations of nodes in a graph to preserve higher-order proximity between nodes. Obtained random walks are then used to train a machine learning model for receiving the final embeddings (94). Node2vec is an algorithmic framework for generating node embeddings, while making a trade-off between breadth-first sampling and depth-first sampling (95). Furthermore, Chen *et al.* proposed HARP, a meta-strategy for improving random-walk based methods like DeepWalk and Node2vec (96).

#### Graph neural networks

Matrix factorization-based methods and random walk-based methods have two serious limitations. First, they cannot generate embeddings for unseen nodes (transductive learning), i.e., a complete re-computation is needed as soon as new nodes appear in the graph. Second, they cannot use features attached to nodes, edges, or graphs. GNNs provide (at least partially) solutions for these limitations (42). They can be divided into graph autoencoders, recurrent GNNs, convolutional GNNs, and spatial-temporal GNNs. Graph autoencoders consist of two parts: an encoder and a decoder. While the encoder reduces the dimensionality of the high-dimensional input, the decoder tries to reconstruct the graph from the embedding. Examples of the neighborhood autoencoders are Deep Neural Graph Representations (DNGR) (97) and Structural Deep Network Embeddings (SDNE) (98). While graph autoencoders can incorporate structural information about a node’s local neighborhood, they are still transductive like random walk-based and matrix-factorization-based methods, and they are computationally expensive due to their fixed input dimension to the autoencoder (42). Recurrent GNNs represent the pioneer works of GNNs and learn node representations with recurrent neural architectures (52). Information between nodes is constantly exchanged until a stable equilibrium is reached. Gated Graph sequence neural networks (99) and graph echo state networks (100) are representatives of recurrent GNNs. The idea of information exchange between nodes motivated recently proposed spatial convolutional GNNs. Convolutional GNNs can be divided into spectral and spatial approaches and provide solutions for the previously mentioned limitations of other graph representation learning techniques. Spectral approaches introduce filters from the perspective of graph signal processing (52). Spatial approaches are based on aggregating information from a node’s local neighborhood (message passing framework) (101). In the message passing framework node features are first transformed using a learnable weight matrix and a non-linear activation function (e.g., sigmoid function) and then aggregated to obtain new node embeddings. The process of transformation and aggregation is repeated *n* times, where *n* is the number of layers. One advantage of spatial convolutional GNNs is that the underlying message passing framework allows to parallelize the operations on each node (i.e., transformation and aggregation) on modern hardware like GPUs to increase computational efficiency. Furthermore, they allow to share trainable parameters (i.e., the learnable weight matrix) between nodes by using the same weight matrix for all node features in one layer which make them statistically and computationally more efficient than spectral approaches. Several different spatial convolutional GNNs were proposed which differ in their architecture, especially their aggregation (e.g., aggregating all neighbors, random walks for aggregation) and pooling (e.g., max-pooling, sum-pooling, average-pooling) parts (101). It is noteworthy that pooling operations need to be permutation-invariant, because of the missing natural order of nodes in a graph (42). GNNs can process homogeneous graphs (i.e., graphs containing only one type of nodes and one type of edges) using homogeneous GNNs (99, 102–105) and heterogeneous graphs (i.e., graphs containing multiple types of nodes and/or edges) using heterogeneous GNNs (106, 107). Spatial-temporal graph neural networks (STGNNs) can consider spatial and temporal dependencies simultaneously, which becomes especially important when analyzing time series on graphs. Therefore, in most cases 1D-Convolutional Neural Networks (CNNs) are combined with graph convolutional layers to learn temporal and spatial dependencies respectively (52). The CNN assigns weights and biases to different timestamps of the time series to consider their importance.

#### Problems and limitations of GNNs

Although GNNs are powerful graph representation techniques, there exist some problems and bottlenecks for them. In the following, we will discuss the problem of over-smoothing, under-reaching and over-squashing in GNNs. Homophily (i.e., nodes that are connected to each other are similar) is an assumption of the message passing framework in GNNs and leads to smoothing of graphs. Smoothness means in this context that node representations become like each other. This smoothness alleviates the classification of nodes in the subsequent prediction step. Over-smoothness occurs when stacking too many layers in a GNN. Consequently, node representations become indistinguishable from each other, worsening the classification accuracy of the GNN (108). Under-reaching occurs when nodes are more than k-hops away from each other, where k is the number of layers in the used GNN, i.e., the nodes are unaware of each other. This is especially important for problems that require long-range information (large problem radius). Adding additional layers to the GNN prevents under-reaching (109). The problem is that adding further layers increases a node’s receptive field (nodes that are k-hops away) exponentially and can thereby lead to over-squashing. Messages from the exponentially growing receptive field are propagated and compressed into fixed-size vectors. Thereby, the graph only learns short-range signals and fails message propagation from distant nodes (109).

#### Other representation learning approaches

There are also some graph representation learning algorithms which cannot be categorized in one of the previous three broad categories. They include popular methods like the large-scale information network embedding (LINE) (110) and GraphGAN (111). LINE can capture first-order and second-order proximity of each node in a graph. It is an embedding technique for handling especially large graphs due to its efficiency (50, 110). GraphGAN consists of a generator and a discriminator, which play a game-theoretical minimax-game. While the generator tries to approximate the ground-truth connectivity distribution of each node in a graph, the discriminator tries to distinguish between the connectivity from ground-truth and the generator (50, 111).

#### Embedding (sub-)graphs

The previously presented approaches (matrix-factorization-based, random walk-based approaches, and GNNs) can generate embeddings for each node in a graph. The obtained node embeddings can also be used to generate (sub-)graph embeddings by aggregating all node embeddings in the (sub-)graph (112). The approaches for aggregating node embeddings can differ, e.g., summing (or averaging) node embeddings (113), or introducing graph coarsening layers (114, 115). Another approach for generating (sub-)graph embeddings is by creating a virtual super-node connected to all nodes that should be included in the (sub-)graph embedding (116). The resulting (sub-)graph embedding can be used for graph-level prediction tasks in graph analytics.

### Graph analytics

After graph representation learning, the obtained low-dimensional embeddings can be used for several graph analytic tasks.

#### Visualization

**Figure 10.**
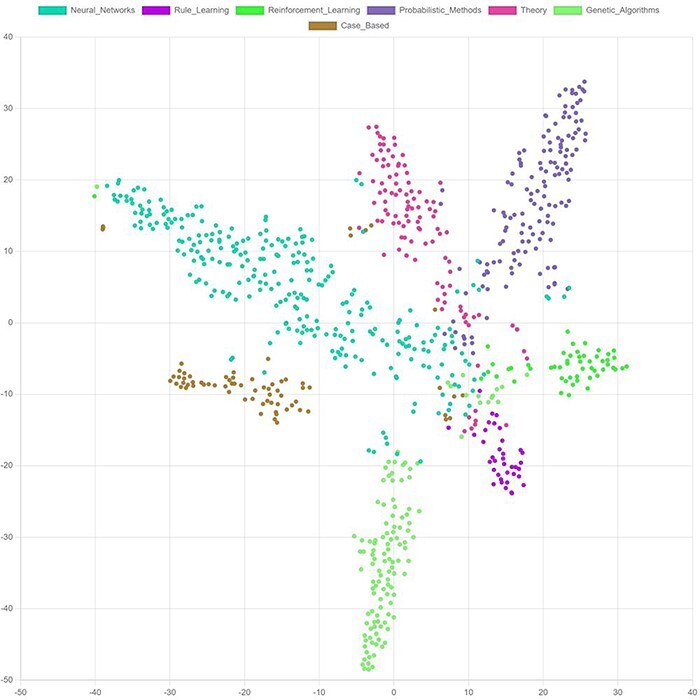
t-SNE. Visualization of final node embeddings after applying two graph convolutional layers on the Cora citation dataset (203) (PyTorch Geometric (204), embedding-dimension: 128). Each color represents a research topic for a node (circle).

Most embedding vectors have between 16 and 128 dimensions, i.e. they cannot be visualized directly in a two-dimensional diagram. Techniques, like t-distributed stochastic neighbor embedding (t-SNE) (117) and principal component analysis (PCA) (118), can be used to reduce the dimensionality of the embeddings and thereby enable visualizing them (Figure 10). Such visualizations help to better understand the model output. It facilitates the identification of outliers or anomalies and allows to identify the margins specific embeddings differ from each other.

#### Graph mining tasks

Besides visualization, there are several other graph analytic tasks that we sum up to graph mining tasks, including link prediction, node classification, (sub-)graph classification, link/node/graph regression, graph generation, node clustering, and network compression. Link prediction aims to find new missing edges (relations) or remove wrong (or in the future disappearing) edges between existing nodes (119, 120). Pairs of node embeddings are used (e.g., by aggregating and linearly transforming the embeddings or by calculating their dot product) to make link predictions (42). In node classification, unlabeled nodes in a graph should be classified based on their features and their position in the graph. This is often a form of semi-supervised learning (i.e., only a subset of nodes has labels) and the goal is to predict the missing labels using the final node embeddings (41). The labeling of (sub-)graphs is accomplished by (sub-)graph classification with (sub-)graph embeddings and provided labels (41). Link/node/graph regression allows the estimation of specific numeric properties of nodes, or (sub-)graphs respectively (41, 121). Graph generation allows the generation of new graphs with specific desired properties and structures (121). Based on the structure and/or properties of nodes, a graph can have multiple different clusters that can be identified with node clustering (e.g., spectral clustering (122)). With the help of network compression, the information stored in a graph is compressed to reduce space requirements (e.g., by utilizing graph embeddings) (52).

## Applications of graph learning

Several different applications of graph learning highlight the potential of utilizing a graph structure and graph learning in various clinical use cases (54, 55). In this section, we classify several clinical applications based on the following graph mining tasks:

- Link prediction (Section 4.1);
- Node classification (Section 4.2);
- (Sub)graph classification (Section 4.3);
- And further graph mining tasks (Section 4.4).

Furthermore, we want to clarify the application of graph learning on a specific use case for a clearer understanding of graph learning (Supplementary Graph learning use case).

### Applications of link prediction

Link prediction aims to find missing or wrong associations between nodes and involves different applications, including modeling drug-drug interactions (43, 123), drug–protein interactions (124), protein–protein interactions (125), disease–disease interactions (126), and knowledge graph completion (46, 92, 127). Drug–drug interactions are used to predict adverse side effects when a patient takes multiple drugs simultaneously (123), and to study drug similarity for inferring novel properties of drugs for drug discovery (128). Use cases of drug-protein networks include predicting drug-target interactions for finding new therapeutic effects of drugs (124). Predicting protein-protein interactions improves our understanding of biological interactions in cells (125). In a comorbidity network (i.e., a graph where diseases are linked if the disease couple affects at least one patient), link prediction is used to predict the onset of future diseases (126). Real-world knowledge graphs widely suffer from incompleteness (92). Therefore, knowledge graph completion is an important task for finding missing relations and inferring new facts (e.g., in medical knowledge graphs). Several different models were proposed for this purpose (47, 129–134). It becomes especially important for dynamic knowledge graphs that change over time (92). Besides predicting missing links in a graph, link prediction can also be used for predicting whether links might disappear in the future (negative links), e.g., in a gene expression or medical referral network (120, 135).

### Applications of node classification

Node classification aims to find labels for unlabeled nodes in a graph. This task is important for population-based datasets, i.e., one graph represents an entire community, and one node represents a single patient. Node classification is used for predicting autism spectrum disorder (136, 137), Alzheimer’s disease (136–139), Parkinson’s disease (138, 139), attention deficit hyperactivity disorder (140), and depression (141). Often imagery data (e.g., functional Magnetic Resonance Imaging (fMRI) data, electroencephalography (EEG) data) and non-imagery data (e.g., gender, age) are used as features attached to individual nodes. In histopathology, node classification was used for segmenting images into diagnostically relevant regions (e.g., for diagnosing prostate cancer) (142). Ma and Zhang proposed AffinityNet for disease type prediction and applied it to cancer genomic data (143). Furthermore, node classification was applied for annotating protein functions (95, 103, 144). Besides, Yue *et al.* classified semantic types of medical terms in a medical term co-occurrence graph (135).

### Applications of (sub-)graph classification

Instead of representing an individual patient as a node with attached features, imagery data from fMRI, EEG, or computer tomography (CT) can be represented as an entire graph. Therefore, data is transferred into a graph structure and graph classification is applied afterwards. Several use cases highlight the applicability of this procedure using EEG data, including seizure prediction (145) and detection (146–148) in epileptic patients, identification of visual stimuli (149), emotional video classification (150), emotion recognition (151–157), and sleep stage classification (158). Furthermore, graph classification was applied to MRI and fMRI data for diagnosing Alzheimer’s disease (159, 160), bipolar disorder (161), autism spectrum disorder (162), early mild cognitive impairment (163), Parkinson’s disease (164), for discovering novel biomarkers (e.g., for neurological disorders) (162, 165), for subject-sex classification (160), for measuring functional connectivity between different brain regions (166), and for the identification of regions of interest in epilepsy networks (167). Histopathological images were represented as a graph to apply GNNs for the identification of lung cancer subtypes (168, 169), breast cancer (171, 172), basal cell carcinoma (173), prostate cancer (174), classification of intestinal glands (175), and predicting lymph node metastasis (176). It was also applied to CT data for COVID-19 induced pneumonia (177) and detecting COVID-19 (177, 178). Finally, graph classification was used in drug discovery for predicting molecular properties (117) and protein interfaces (179).

### Applications of further graph mining tasks

Besides applications for the previously mentioned most prominent graph mining tasks, there are further ones for other graph mining tasks. For example, node regression was applied for predicting the brain age of human subjects (179). The gap between the estimated brain age and the true (chronological) age is important for understanding biological pathways relevant to aging, assessing risks for brain disorders, and developing new therapies. Another application of graph learning is learning the graph structure of Electronic Health Records to improve the performance of downstream prediction tasks, e.g., heart failure prediction (180). Graph regression was applied for predicting survival outcomes for glioma and clear cell renal carcinoma (181), and for determining colon cancer stage (182) in histopathology. Furthermore, Sureka *et al.* proposed an approach for modeling histology tissue as a graph of nuclei and used convolutional GNNs for visualization and diagnosis of diseases like breast and prostate cancer (183). Another graph mining task is module (or subgraph) identification in biological graphs (e.g., protein–protein or gene co-expression networks) which helps to better understand and treat diseases (184, 185). Finally, graph learning was also used for recommending medication combinations for patients with complex health conditions (186, 187).

## Conclusion and future directions

In this paper, we reviewed current state-of-the-art approaches for storing and analyzing datasets with the help of an underlying graph structure. A graph structure is especially useful for representing interconnected data like clinical data. In this context, we highlight several existing applications that exploit the potential of using graphs and graph learning approaches. Furthermore, we classified these applications according to the used graph mining tasks (link prediction, node classification, (sub-)graph classification). This classification is important to find an appropriate graph learning algorithm for the desired use case. It is noteworthy that sometimes one can transfer one or multiple graphs with a specific graph mining task to another graph mining task, e.g., by transferring a single patient node with features (node classification) into a graph with features as nodes (graph classification). The question one needs to ask in advance is which data representation and therefore which graph mining task performs better for a specific use case.

Currently, each application has its individual method (e.g., for data pre-processing, and graph generation) even for the same kind of data (e.g., fMRI data). A generic framework for transferring clinical data into a graph and applying graph learning afterwards, would help to solve several scientific issues quicker and to make the methodology more reproducible. Furthermore, scientists could use previously trained models as a foundation for their use case (transfer learning). Currently, the implementation of such a generic framework is especially difficult due to diverse data formats and the large heterogeneity of clinical data.

Modern graph representation learning techniques such as GNNs consider the input features and thereby decide which features are more important than others. An interesting question might be how much features that are automatically extracted by tools like GNNs differ from features selected by domain experts.

Several advantages of graph databases facilitate storage, retrieval, and exploration of data. First, graph databases provide a higher flexibility in their data schema than relational databases (188). Second, graph query languages like Cypher prevent the formulation of complex Join operations. Third, the easy visualization of complex interconnected graph data (e.g., with Neo4j Bloom (189)) provides a comprehensive overview about the underlying data.

Currently, there are only limited advances in applying machine learning algorithms directly on a graph database, e.g., Neo4j’s graph data science library (190) and TigerGraph’s data science library (191). Furthermore, to the best of our knowledge, there is no framework that can process graph data from various graph databases with modern graph learning approaches. This strategy is quite promising, because querying only the necessary data might increase the performance for extremely large datasets. Such a graph learning framework could standardize and unify the various query languages of different graph databases (e.g., Cypher and GSQL), integrate state-of-the-art deep learning frameworks like PyTorch (192), TensorFlow (193), Deeplearning4j (194), Microsoft CNTK (195), or flux (196), and allow clear visualizations. A framework using relational databases already exists (197) and showed the potential for easy application of machine learning algorithms and visualizations of the results (198).

However, there are still some other limitations in using graph databases and graph learning algorithms. First, relational databases are much more common in industry making them preferred over graph databases due to their decades-long utilization. Some graph learning algorithms have serious limitations in their performance, and some cannot use features attached to nodes/edges/graphs. Although GNNs seem promising for improving computation efficiency and feature utilization, there are still some problems like over-smoothing, underreaching and over-squashing. Finally, missing standards, diverse formats, lexical disparities, class imbalances, data privacy and the enormous heterogeneity of clinical data makes applying machine learning algorithms even more complex. A combination of implementing further graph learning applications, establishing swarm learning (199) and standardizing data and formats will help to overcome the mentioned problems.

## Supplementary Material

baad045_Supp

## Data Availability

All required material is contained in the Supplementary material.
