# Correction to: The importance of graph databases and graph learning for clinical applications

**DOI:** 10.1093/database/baae006

**Published:** 2024-01-20

This is a correction to: Daniel Walke, Daniel Micheel, Kay Schallert, Thilo Muth, David Broneske, Gunter Saake, Robert Heyer, The importance of graph databases and graph learning for clinical applications, Database, Volume 2023, 2023, baad045, https://doi.org/10.1093/database/baad045

In the originally published version of this manuscript, the in-text citation of reference 64 was inadvertently omitted. Due to this error, the numbering of subsequent in-text citations was also incorrect.

This has been corrected.

