# Supplementary material for: The importance of graph databases and graph learning for clinical applications
Source: Database (Oxford). 2023 Jul 10;2024:baad045. doi: 10.1093/database/baad045 (PMC10332447; doi:10.1093/database/baad045)
Supplement: baad045_Supp [file baad045_supp.zip › suppl_data/Graph learning use case.docx]

### Graph learning use case

We will introduce a simple use case for applying graph learning algorithms for an easier understanding. Consider a simple undirected graph consisting of n nodes (patients), which are connected via edges based on some kind of similarity (e.g., same age, direct family members) (fig. 1). All patients have a feature vector attached containing information like blood pressure, blood sugar, gender, and age. Additionally, most patients have a defined class label regarding their health status (i.e., diabetes or no diabetes). The goal is to predict the health status for the unlabeled patients. To find the missing labels, one tries to find good node representations. The basic idea is that similar nodes (e.g., with the same label) have similar representations. Although patients with an underlying disease might have highly variable disease patterns, node representations often still differ from representations of healthy patients. By finding good node representations, we can infer which nodes might be similar and therefore might have the same label/diagnosis. This task is called node classification.


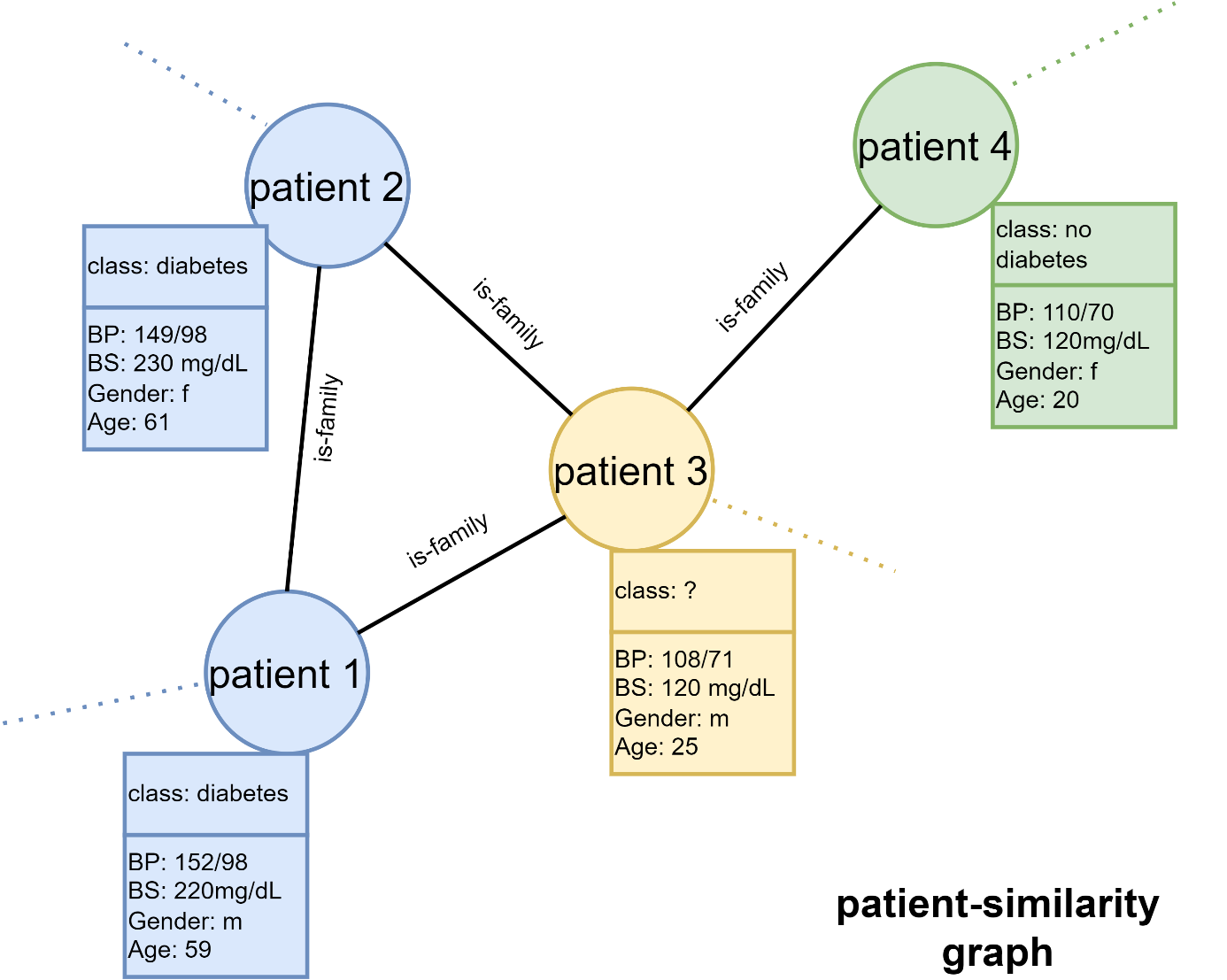


Figure 1: Example of an undirected graph with nodes (patients) and connecting edges (direct family members). Each node has features (BP: blood pressure, BS: blood sugar, gender, and age) and a class label (diabetes, no diabetes, unknown) attached. One goal might be to predict missing class labels (node classification).

**Manual feature extraction.** If we are only using manual feature extraction, we first need to define what features we want to extract. In this case, we want to extract node-level features because the task is node classification. This means that we could use measures like node degree, node centrality and the clustering coefficient. The node degree counts the number of neighbors of each node. Node centrality measures each node’s importance, e.g., by summing up the shortest paths to each node (closeness centrality). Finally, clustering coefficients checks how tightly clustered each node is by measuring the connections between a node’s neighbors.

**Matrix-factorization and random walk-based methods.** Matrix-factorization-based methods and random walk-based methods only use node connections to find good representations. This is either done by factorizing a similarity matrix or by randomly walking through the graph. For matrix-factorization-based methods, we first define a similarity matrix, e.g., by directly using the connecting edges (adjacency matrix). Then, we factorize this matrix, e.g., we try to find node representations such that the scalar product of the node representation of a node and the transposed representation of another node approximates the respective entry in the similarity matrix. The intuition behind matrix factorization is that two nodes have similar representations if they have a high similarity according to the used matrix. For random walk-based methods, we choose a node and then randomly pick a neighboring node. The last step is repeated until the desired random walk length is obtained. Finally, the obtained random walks for each node are used for obtaining the final representations. The intuition is that two patient nodes have similar representations if they co-occur on many short random walks (i.e., are somehow connected).

**Graph Neural Networks.** The previously mentioned approaches only incorporate topological information, i.e., a node’s position in the graph. This is mostly insufficient especially for diagnosing patients because important information is stored as features attached to nodes. If we are using a convolutional graph neural network (GNN), we can use the features attached to the patient nodes. In a GNN a node collects information from his neighborhood (also called information propagation, or message passing). The depth of a GNN defines how large the neighborhood is. If the depth is one, a node only collects information from its direct neighbors, if the depth is two it collects information from its direct neighbors and from the direct neighbors of each neighbor and so on. The intuition is that nodes have similar representations if they are connected and have similar features. Recurrent GNNs do not use depth, instead they repeat the information propagation until a stable equilibrium for all nodes is reached. Graph autoencoders are not applicable, because they are mainly used for graph generation methods, and spatial-temporal GNNs are only applicable for time series.
